# Immune checkpoint inhibitors and endocrinopathies in pediatric brain tumor patients

**DOI:** 10.1515/jpem-2024-0243

**Published:** 2024-12-16

**Authors:** Carly R. Westermann, Tom B. Davidson, Kaaren Waters, Ashley S. Margol, Clement C. Cheung

**Affiliations:** Frank H. Netter MD School of Medicine at Quinnipiac University, North Haven, CT, USA; Cancer and Blood Disease Institute and Division of Hematology and Oncology, Children’s Hospital Los Angeles, Los Angeles, CA, USA; and Department of Pediatrics, Keck School of Medicine, University of Southern California, Los Angeles, CA, USA; Cancer and Blood Disease Institute and Division of Hematology and Oncology, Children’s Hospital Los Angeles, Los Angeles, CA, USA; Cancer and Blood Disease Institute and Division of Hematology and Oncology, Children’s Hospital Los Angeles, Los Angeles, CA, USA; and Department of Pediatrics, Keck School of Medicine, University of Southern California, Los Angeles, CA, USA; The Division for Endocrinology, Diabetes, and Metabolism, Children’s Hospital Los Angeles 4650 Sunset Blvd, MS#61 90027, Los Angeles, CA, USA; and Department of Pediatrics, Keck School of Medicine, University of Southern California, Los Angeles, CA, USA

**Keywords:** brain tumor, endocrine diagnosis, immune-related adverse events, immune checkpoint inhibitors, pediatrics

## Abstract

**Objectives:**

Immune checkpoint inhibitors (ICIs) are emerging treatment options for children with brain tumors, who are already at risk for developing endocrinopathies due to tumor location and treatment. Endocrine ICI-related adverse effects (irAEs) are common in adults but poorly characterized in the pediatric population. The aims of this study were to determine in pediatric brain tumor patients in a single institution (1) if endocrine surveillance took place before and after ICIs were initiated, and (2) the occurrence of endocrine irAEs.

**Methods:**

This is a retrospective chart review of 22 pediatric brain tumor patients treated with ICIs at Children’s Hospital Los Angeles between 2010 and 2022. We analyzed endocrine laboratory results, patient demographics, and treatment course.

**Results:**

Most patients (82 %) received surveillance in at least one endocrine system before ICI treatment – all had thyroid function tested (100 %) whereas non-thyroid endocrine functions were seldomly assessed (6–22 %). Only those patients with surveillance prior to treatment had ongoing surveillance after ICI initiation – 100 % for thyroid function and 17–39 % for other endocrine systems. Hypothyroidism was the only endocrine problem diagnosed after ICI initiation, in two patients (9 %). Of note, most patients (68 %) expired during or shortly after ICI treatment.

**Conclusions:**

This is one of the first institutional surveys of pediatric ICIs in a high-volume pediatric brain tumor center. Thyroid surveillance commonly occurred in pediatric patients, revealing diagnoses of hypothyroidism, which is consistent with adult data. However, little information is available for non-thyroid endocrine conditions, reflecting the need for comprehensive and systematic endocrine surveillance.

## Introduction

Pediatric brain tumors are the most common solid childhood tumors and have the highest mortality rates [1, 2]. Brain tumors are especially challenging to treat due to their location and biological characteristics [3]. Immune checkpoint inhibitors (ICIs) are an emerging treatment modality for difficult-to-treat or refractory adult cancers, and, as such, are now being used in children with brain tumors that are similarly unresponsive to conventional treatment [4–6]. Briefly, naturally occurring immune checkpoint proteins negatively regulate the immune system to ensure self-reactive T-cells are eliminated in order to prevent autoimmunity. It is known that tumor cells utilize immune checkpoints to evade destruction by the immune system via various mechanisms [7]. ICIs are monoclonal antibodies that target negative regulatory pathways of the natural immune response, specifically cytotoxic T-lymphocyte-associated antigen 4 (CTLA-4) such as ipilimumab; programmed cell death protein-1 (PD-1), such as cemiplimab, nivolumab, and pembrolizumab; and the ligand PD-1L such as durvalumab [7]. By inhibiting such signaling, ICIs thereby restore the natural anti-tumor response by the immune system.

The use of ICIs has risen dramatically as they have shown efficacy in many adult cancers [8–12]. However, given their mechanism of action, ICIs may lead to the development of immune-related adverse events (irAEs) due to autoimmunity and/or autoinflammation. One of the most commonly reported irAEs in the adult population is endocrine dysfunction, which includes thyroid disease, primary adrenal insufficiency, hypophysitis, and insulin-deficient diabetes mellitus [13–16]. Recent studies have reported the incidence of endocrinopathies in adults treated with ICIs to range from 5–20 % [17, 18], and guidelines for their management have begun to be developed [15, 19, 20]. In contrast, endocrine irAEs in the pediatric population are not well characterized as their description has been limited mostly to anecdotal reports or reviews [21–25].

Endocrine late effects that stem from disruption of the hypothalamic-pituitary-endocrine axes are common in children with brain tumors [26]. Endocrinopathies from ICIs add further complexity to identifying and treating endocrine adverse events that survivors of childhood brain cancers develop. The goals of this study were (1) to evaluate a single-center frequency of surveillance for endocrinopathies in pediatric brain tumor patients treated with ICIs and (2) to describe the occurrence of such endocrinopathies. This study is one of the first institutional surveys in a high-volume pediatric brain tumor clinic and will add to the limited literature on this topic in a pediatric population and thus better inform surveillance and timely referral and treatment for children with brain tumors.

## Methods

### Study design

This was a retrospective cohort study that included all children who were diagnosed with a brain tumor and treated with an ICI at Children’s Hospital Los Angeles between 2010 and 2022. The neuro-oncology database was reviewed to identify all patients that received ICIs under Children’s Oncology Group (COG) protocols within the above time period, yielding 22 patients to be included in the study. The (COG) trials included PBTC 45, PNOC013, BMS209908, PNOC19. ICIs included in this study were anti-CTLA-4 (ipilimumab), anti-PD-1 (cemiplimab, pembrolizumab, nivolumab) and anti-PD-L1 (durvalumab). The study protocol and data collection forms were reviewed and exempted by the Children’s Hospital Los Angeles Institutional Review Board.

### Definitions

Endocrine surveillance in the “before” group was defined as hormonal testing performed within 3 months prior to the initiation of ICI treatment. The “after” group represents testing after the initiation of ICI treatment, which encompasses time during active treatment and any time after ICI treatment was stopped. For patients with ongoing surveillance at the time of data collection, the date of the most recent endocrine lab result was considered. Endocrine surveillance was evaluated by the following systems: (1) thyroid – thyroid-stimulating hormone (TSH), free T4, and total T3; (2) adrenal – ACTH and cortisol; (3) reproductive – pediatric luteinizing hormone (LH), pediatric follicle-stimulating hormone (FSH), and estradiol (females) or testosterone (males); (4) pituitary – insulin-like growth factor1 (IGF-1), IGF binding protein-3 (IGFBP-3); and prolactin; (5) diabetes mellitus-plasma glucose and hemoglobin A1c (HbA1c). Patients often had plasma glucose measured with other metabolic panels; therefore, glucose was only considered in the context of concurrent HbA1c testing.

The duration of ICI treatment was calculated as the number of days from the first day of treatment to the last day of treatment or date of patient death. Some patients received multiple ICIs, and, in those cases, the date of the first ICI received was reported.

### Outcomes

Data collected included patient sex, age at diagnosis, ethnicity; type of brain tumor; previous surgery, radiation, chemotherapy; the ICI agent(s) used and duration of treatment; endocrine lab results at baseline and after ICI initiation; endocrine diagnoses; and patient survival status. The primary outcome measures were frequency of endocrine surveillance before and after ICI initiation. Secondary outcomes included endocrine diagnoses.

## Results

A total of 22 patients were included in this study. The baseline patient demographics and clinical characteristics are shown in Table 1. The majority of patients were male (59.1 %, n=13) and Hispanic or Latino (54.5 %, n=12). The mean age at diagnosis was 7.6 years, with a range of 0.7–17 years. The most common tumors were anaplastic astrocytoma (27.3 %, n=6), glioblastoma (22.8 %, n=5), ependymoma (18.2 %, n=4), and diffuse intrinsic pontine glioma (18.2 %, n=4). Table 2 shows that most patients had been treated surgically (68.2 %, n=15), and received radiation (95.5 %, n=21) and chemotherapy (68.2 %, n=15) prior to ICI treatment. No patients had prior endocrine diagnoses before ICI treatment. Pembrolizumab was the most commonly used ICI (40.9 %, n=9), followed by durvalumab (27.2 %, n=6), cemiplimab (22.7 %, n=5), nivolumab (4.5 %, n=1), and combined nivolumab and ipilimumab (4.5 %, n=1). The range of duration of ICI treatment was 14–1,072 days.

Among the cohort, 20 patients discontinued ICI at the end of the study period due to tumor progression (60.0 %, n=12), adverse reactions (15.0 %, n=3), or due to expiration during treatment (15.0 %, n=3) (Supplemental Table 1). Notably, the majority of patients expired (66.7 %, n=14). At the time of analysis, two patients were still receiving ICI treatment (Supplemental Table 1).

Of the 22 patients, the majority (81.8 %, n=18) received endocrine surveillance of at least one system before ICI treatment; however, only those same 18 patients had ongoing surveillance per treatment protocol or through referral to endocrinology after ICI initiation (Table 3). Among these patients, all of them had thyroid surveillance, specifically TSH (100 %, n=18) and free T4 (94.4 %, n=17) (Table 4). No patients had total T3 checked. Non-thyroid laboratory surveillance before treatment was limited, with only two patients checked for adrenal function, three for reproductive hormones, four for growth-related analytes, one for hypophysitis/abnormal prolactin, and one for diabetes. Thyroid surveillance continued among all 18 patients after ICI initiation (100 %), while non-thyroid surveillance continued to be limited seven patients were checked for adrenal function, six for reproductive hormones, six growth related analytes, three for prolactin, and four for diabetes mellitus.

Two patients (4.3 %) were diagnosed with hypothyroidism after ICI treatment. No other endocrine diagnoses were made.

## Discussion

Immune checkpoint inhibitors have improved survival rates for many types of adult cancers and are increasingly being used in pediatric cancers. ICIs promote T-cell-mediated anti-tumor responses, and, correspondingly, increase the risk of autoimmunity as an adverse event. Identified risk factors associated with developing endocrine irAEs include the type of ICI used, higher doses, and combination therapy; whereas the risks associated with duration, cancer type, and patient-specific factors have not been well-studied [27–29]. Guidelines for surveillance of endocrine irAEs currently exist only for adults [15, 19, 30–32]. Currently no guidelines exist for pediatric patients similarly treated. This study reports that pediatric brain tumor patients treated with ICIs do not commonly have endocrine surveillance, except for thyroid testing.

Thyroid dysfunction is the most frequently reported endocrine irAE in adults receiving ICIs, occurring in approximately 10 % of patients treated with anti-PD-1 or anti-PD-LI therapy and in up to 20 % of those treated with combination therapy [13]. The most common patterns of thyroid dysfunction are transient thyrotoxicosis that develops into hypothyroidism [33, 34]. Of note, patients with pre-existing anti-thyroid antibodies have been found to be at an increased risk for thyroid dysfunction when being treated with ICIs [35, 36]. Pediatric brain tumor patients, independent of ICI treatment, are known to be at a high risk for primary and central hypothyroidism due to late effects of treatment, in particular whole brain or craniospinal radiation [37]. Although this adds to the complexity of delineating ICI-mediated endocrinopathies from late effects of non-ICI cancer treatment, it does not negate the ongoing need for continued thyroid surveillance in this population. Given the high incidence of thyroid irAEs in adult patients, the recommendation is to monitor thyroid function tests frequently during ICI treatment, which have influenced clinical trial requirements for testing [19, 38, 39]. In our study, all our patients were on COG protocols that require thyroid function testing pre-treatment, regularly throughout treatment (each cycle to every fourth cycle), and post-treatment. This accounts for the 100 % occurrence of thyroid surveillance in our study among patients who received surveillance. Consistent with the adult literature, of the 22 pediatric patients included in our study, two patients (9.1 %) were diagnosed with hypothyroidism after the initiation of ICI treatment.

Case reports and reviews in adults report a low incidence (1.0 % or less) of ICI-related type I diabetes mellitus (T1DM) but highlight the rapid onset of diabetic ketoacidosis (DKA) and pancreatitis which these patients often initially present [40–42]. Two pediatric patients presenting with DKA due to ICI-related T1DM have been reported [22, 24]. As the report by Dasgupta et al. notes, ICI-related T1DM differs from the spontaneous childhood autoimmune disease in that ICI-related T1DM often occurs rapidly and may not present with an elevated HbA1c. Additionally, a systemic review reported only 53 % of patients with ICI-mediated DM having positive autoantibodies, possibly related to the rapid onset, or the occurrence only in the context of ICI treatment [42]. This led to the recommendation that the evaluation of hyperglycemia in ICI-treated patients should include measurement of c-peptide, as their patient also did not have positive autoantibodies [22]. Only one patient in our study had HbA1c and glucose measurements before ICI treatment, four were checked after, and none had c-peptide measured.

Endocrine clinical presentations and evaluations in pediatric patients may differ greatly from those in adults for whom the existing guidelines are designed. Recent studies have demonstrated that adult-onset T1DM is often less acute, has different requirements for initial insulin therapy compared to childhood-onset, and has different genetic risk factors [43–45]. Such variations in underlying autoimmunity among different age groups may predispose children undergoing ICI treatment to different profile of endocrine irAEs than are seen in adults.

The patient reported by Dasgupta et al., also tested positive for 21-hydroxylase autoantibodies, which is indicative of autoimmune adrenalitis. Adrenal insufficiency is a rare but life-threatening endocrine irAE that is limited to a few adult case studies, and more often is reported as having a central basis in the context of ICI-related hypophysitis [17, 40, 46, 47]. Guidelines exist that recommend monitoring a baseline morning serum cortisol in the context of breast cancer treatment and is proposed as a consideration during ICI treatment for patients with pre-existing endocrine disease [20, 48]. Serum cortisol levels were assessed in two members (11.1 %) of our cohort before ICI treatment and in six (33.3 %) after, with no abnormalities found. In regard to hypophysitis as described in adults, it has been found to occur more commonly with anti-CTLA-4 agents (ranging from 3.0–10.0 %) and combination therapy (approximately 7.0 %), but no pediatric cases have been reported thus far [17, 30, 49].

The developmental needs of children also require unique considerations with respect to endocrine surveillance. For example, it is unlikely that adults will be assessed for pubertal or growth disorders, but these are high priority concerns for developing children whereas reproductive dysfunction is more relevant in the adults. Survivors of childhood cancer, especially brain tumors, face long-term endocrine sequalae independent of ICI treatment [37, 50]. Currently, the existing adult guidelines do not emphasize growth surrogate markers in their recommendation for monitoring, during or after ICI treatment.

There are several limitations to this study. First, the retrospective design subjects our study to missing and confounding data and the potential for misclassification. This can be remedied by prospective studies with larger cohorts of patients and deliberate data collection. Second, although our results are derived from an institution that sees a high volume of potentially eligible patients compared to others due to its combined neuroendocrinology-neuro-oncology clinic, the number of patients included in this study remained low. Multi-site studies in brain tumor patients may allow us to truly delineate the extent of endocrinopathy in pediatric ICI treatment. Third, ICIs were typically used late in the treatment course of all patients. Until there are sufficient data to suggest that ICIs should be used as first-line treatments for pediatric brain tumors, it will remain difficult to assess a definite causal relationship between ICIs and specific endocrinopathies. As ICIs are often used to treat pediatric brain tumor patients with advanced and refractory disease, mortality rates were high, and limited our ability to assess the evolution of ICI-associated endocrinopathies over time. Finally, brain tumors themselves (depending on their location) and their treatments (e.g., radiation) are confounding factors for the development of endocrinopathies, with a nationwide cohort study reporting 22.1 % of non-ICI-treated childhood brain tumor survivors developing at least one endocrinopathy within 5 years after diagnosis [51]. As such, any endocrinopathy that occurs during ICI treatment may not be due solely to the ICI exposure, but rather be a late effect of earlier or concomitant non-ICI treatment [50–52]. Expanding future studies to include non-brain tumor patients treated with ICIs would allow for better control of these factors in assessing the casual relationship between ICIs and the development of specific endocrinopathies.

## Conclusions

This is one of the first institutional reports in a high-volume pediatric brain tumor clinic reporting on the prevalence of endocrine surveillance and occurrence of endocrine irAEs in children treated with ICIs. This study revealed that thyroid function is commonly surveyed, however, other endocrinopathies that occur in adults are not part of current pediatric protocols for monitoring during ICI treatment. Further prospective studies are needed to truly capture the incidence of pediatric endocrinopathy during ICI treatment and create appropriate recommendations for this population.

## Supplementary Material

Supplementary Material

## Figures and Tables

**Table 1:** Patient demographics.

Characteristic		n, %

Sex	Male	13 (59.1)
	Female	9 (40.9)
Age at diagnosis, mean (range)	7.6 (0.7–17)	
Ethnicity	Hispanic or Latino	12 (54.5)
	Not Hispanic or Latino	8 (36.4)
	Unknown	2 (9.1)
Patient status at the end of the study	Alive	7 (31 .8)
	Expired	15 (68.2)
Type of cancer	Astrocytoma	6 (27.3)
	DIPG^a^	4 (18.2)
	Ependymoma	4 (18.2)
	Glioblastoma	5 (22.8)
	Gliosarcoma	1 (4.5)
	High-grade glioma	1 (4.5)
	Medulloblastoma	1 (4.5)

a DIPG, diffuse intrinsic pontine glioma.

**Table 2:** Patient treatment details.

Characteristic		n, %

Prior surgical treatment	Yes	15 (68.2)
	No	7 (31 .8)
Prior radiation treatment	Yes	21 (95.5)
	No	1 (4.5)
Prior chemotherapy treatment	Yes	15 (68.2)
	No	7 (31 .8)
Endocrine diagnosis before ICI	Yes	0 (0.0)
	No	22 (100.0)
Initial ICI agent	Cemiplimab	5 (22.7)
	Durvalumab	6 (27.2)
	Pembrolizumab	9 (40.9)
	Nivolumab	1 (4.5)
	Nivolumab + ipilimumab	1 (4.5)
Duration of ICI in days (range)		14–1,072
Discontinued ICI during study	Yes	20 (90.9)
period	No	2 (9.1)

**Table 3:** Status of endocrine surveillance.

Surveillance received?	Before ICI		During/after ICI	
	Number of patients	% of patients (n=22)	Number of patients	% of patients (n=22)

Yes	18	81.8	18	81.2
No	4	18.2	4	18.2

**Table 4:** Endocrine surveillance by system among patients who received surveillance before and during/after ICI treatment.

System	Laboratory data	Before ICI		During/after ICI	
		Number of patients	% of patients (n=18)	Number of patients	% of patients (n=18)

Thyroid	TSH	18	100.0	18	100.0
	Free T4	17	94.4	18	100.0
	Total T3	4	22.2	4	22.2
Adrenal	Cortisol	2	11.1	7	38.9
	ACTH	1	5.6	0	0.0
Reproductive	FSH	3	16.7	6	33.3
	LH	2	11.1	6	33.3
	Estradiol	0	0.0	1	5.6
	Testosterone	2	11.1	4	22.2
Pituitary	IGF-1^a^	4	22.2	6	33.3
	IGFBP-3^b^	4	22.2	6	33.3
	Prolactin	1	5.6	3	16.7
Diabetes	Glucose	1	5.6	4	22.2
	HbA1c	1	5.6	4	22.2

a IGF-1, insulin-like growth factor-1;

b IGFBP-3, IGF binding protein-3.

## Data Availability

All data generated or analyzed during this study are included in this article. Further enquiries can be directed to the corresponding author.
