# Supplementary material for: Immune checkpoint inhibitors and endocrinopathies in pediatric brain tumor patients
Source: J Pediatr Endocrinol Metab. Author manuscript; Available in PMC 2025 Feb 17. (PMC11832116; doi:10.1515/jpem-2024-0243)
Supplement: Supplementary Material [file NIHMS2045082-supplement-Supplementary_Material.docx]

| **Patient ID** | **Age at Diagnosis (years)** | **Duration of ICI Treatment (days)** | **Discontinued ICI during Study Period?** | **Reason for Discontinuation of ICI during Study Period** | **Patient Status at End of Study Period** |
| --- | --- | --- | --- | --- | --- |
| 1 | 7 | 140 | Yes | Tumor progression | Expired |
| 2 | 14 | 195 | Yes | Tumor progression | Expired |
| 3 | 1 | 14 | Yes | Adverse reaction | Expired |
| 4 | 4 | 182 | Yes | Tumor progression | Expired |
| 5 | 15 | 42 | Yes | Tumor progression | Expired |
| 6 | 11 | 41 | Yes | Tumor progression | Expired |
| 7 | 8 | 166 | Yes | Tumor progression | Alive |
| 8 | 17 | 468 | Yes | Adverse reaction | Alive |
| 9 | 0.7 | 42 | Yes | Tumor progression | Alive |
| 10 | 12 | 94 | Yes | Expired during treatment | Expired |
| 11 | 9 | 1072 | Yes | Tumor progression | Expired |
| 12 | 3 | 329 | Yes | Completed treatment | Expired |
| 13 | 2 | 239 | Yes | Tumor progression | Alive |
| 14 | 16 | 93 +^a^ | No | – | Alive |
| 15 | 4 | 184 +^a^ | No | – | Alive |
| 16 | 3 | 42 | Yes | Adverse reaction | Expired |
| 17 | 1 | 719 | Yes | Completed treatment | Alive |
| 18 | 16 | 72 | Yes | Tumor progression | Expired |
| 19 | 4 | 40 | Yes | Expired during treatment | Expired |
| 20 | 11 | 103 | Yes | Tumor progression | Expired |
| 21 | 3 | 15 | Yes | Expired during treatment | Expired |
| 22 | 12 | 24 | Yes | Tumor progression | Expired |

^a^Patients had ongoing treatment at end of study period and duration of treatment reflects number of days until end of data collection
